# Increasing Resistance to Azithromycin in Neisseria gonorrhoeae in Eastern Chinese Cities: Resistance Mechanisms and Genetic Diversity among Isolates from Nanjing

**DOI:** 10.1128/AAC.02499-17

**Published:** 2018-04-26

**Authors:** Chuan Wan, Yang Li, Wen-Jing Le, Yu-Rong Liu, Sai Li, Bao-Xi Wang, Peter A. Rice, Xiao-Hong Su

**Affiliations:** a STD Clinic, Institute of Dermatology, Chinese Academy of Medical Sciences and Peking Union Medical College, Nanjing, Jiangsu, China; b Division of Infectious Diseases and Immunology, University of Massachusetts Medical School, Worcester, Massachusetts, USA

**Keywords:** Neisseria gonorrhoeae, azithromycin, antimicrobial resistance, molecular epidemiology, eastern Chinese cities

## Abstract

Azithromycin resistance (AZM-R) of Neisseria gonorrhoeae is emerging as a clinical and public health challenge. We determined molecular characteristics of recent AZM-R Nanjing gonococcal isolates and tracked the emergence of AZM-R isolates in eastern Chinese cities in recent years. A total of 384 N. gonorrhoeae isolates from Nanjing collected from 2013 to 2014 were tested for susceptibility to AZM and six additional antibiotics; all AZM-R strains were characterized genetically for resistance determinants by sequencing and were genotyped using N. gonorrhoeae multiantigen sequence typing (NG-MAST). Among the 384 isolates, 124 (32.3%) were AZM-R. High-level resistance (MIC, ≥256 mg/liter) was present in 10.4% (40/384) of isolates, all of which possessed the A2143G mutation in all four 23S rRNA alleles. Low- to mid-level resistance (MIC, 1 to 64 mg/liter) was present in 21.9% (84/384) of isolates, 59.5% of which possessed the C2599T mutation in all four 23S rRNA alleles. The 124 AZM-R isolates were distributed in 71 different NG-MAST sequence types (STs). ST1866 was the most prevalent type in high-level AZM-R (HL-AZM-R) isolates (45% [18/40]). This study, together with previous reports, revealed that the prevalence of AZM-R in N. gonorrhoeae isolates in certain eastern Chinese cities has risen >4-fold (7% to 32%) from 2008 to 2014. The principal mechanisms of AZM resistance in recent Nanjing isolates were A2143G mutations (high-level resistance) and C2599T mutations (low- to mid-level resistance) in the 23S rRNA alleles. Characterization of NG-MAST STs and phylogenetic analysis indicated the genetic diversity of N. gonorrhoeae in Nanjing; however, ST1866 was the dominant genotype associated with HL-AZM-R isolates.

## INTRODUCTION

Gonorrhea is the second most prevalent sexually transmitted bacterial infection in the world. In 2012, the WHO estimated that the number of new cases of gonococcal infection worldwide was 78.3 million per year (1). In 2016, the Chinese Centers for Disease Control and Prevention reported 115,024 cases; gonorrhea ranked fifth among reported infectious diseases in China (2).

Antimicrobial resistance (AMR) of Neisseria gonorrhoeae is a major public health problem worldwide. N. gonorrhoeae has developed AMR to most antimicrobial agents previously recommended for treatment (3). The emergence of decreased susceptibility or frank resistance of N. gonorrhoeae to ceftriaxone (3–5) (the last available agent used for first-line empirical monotherapy) has resulted in public health recommendations from Australia, Canada, Europe and the United States to use dual antimicrobial therapy consisting of a single dose of ceftriaxone (250 mg or 500 mg intramuscularly) and azithromycin (AZM) (1 g or 2 g orally) together (6–9). In addition to treating gonorrhea alone, AZM is also used to treat presumptive coinfection with Chlamydia trachomatis (6, 10, 11), thereby achieving *de facto* dual therapy for gonococcal infection. Incremental increases in gonococcal MICs of AZM have been reported from several continents (12–14). The sporadic development of high-level AZM resistance (HL-AZM-R) (15–22) and numerous clinical failures (23–27) in many countries are of great concern and threaten the long-term efficacy of the currently recommended regimen.

AZM exerts its antimicrobial effect by binding to the 23S rRNA component of the 50S ribosome and inhibits protein synthesis by preventing the elongation of peptide chains. AZM resistance (AZM-R) in N. gonorrhoeae results from point mutations in 23S rRNA alleles of the *rrl* gene that include A2143G (numbering corresponds to A2059 in Escherichia coli) or C2599T (numbering corresponds to C2611T in E. coli) and the overexpression of the MtrCDE efflux pump caused by mutations either in the coding region of the *mtrR* gene or in the *mtrR* promoter (28–31). In addition, mutations in the ribosomal genes *rplD* (encoding ribosomal protein L4) and *rplV* (encoding ribosomal protein L22), as described for E. coli and Streptococcus pneumoniae, may confer resistance to macrolides due to a change in the conformation of 23S rRNA domains (32); however, this mutation is unusual in N. gonorrhoeae (3).

China developed a national surveillance system to monitor N. gonorrhoeae AMR in 1987 and then joined the WHO Western Pacific Regional Gonococcal Antimicrobial Susceptibility Programme in 1992. However, AZM resistance had not been monitored at the national level in China before 2013. Consequently, AZM resistance and molecular characterization of AZM-R N. gonorrhoeae in China had been limited (22, 33–35). Between 2013 and 2016, the China Gonococcal Resistance Surveillance Programme (China-GRSP) determined that the prevalence of gonococcal AZM-R was 18.6% nationally (3,849 isolates tested from 7 sentinel sites located throughout China) (36). In the absence of new antimicrobials available for the treatment of gonorrhea, classification of the emergence and dynamics of AZM-R N. gonorrhoeae strains on a regional and national basis is important in order to update treatment recommendations successfully. Accordingly, since 2013, AZM has been added to antimicrobial susceptibility panels to test N. gonorrhoeae susceptibility in Nanjing, China. Here we report the prevalence of AZM-R N. gonorrhoeae and the genetic characteristics of AZM-R isolates isolated in Nanjing from January 2013 to December 2014, and we track chronologically the emergence of AZM-R N. gonorrhoeae isolates in eastern Chinese cities over the past several years.

## RESULTS

### Subject characteristics.

Among 905 consecutively enrolled male subjects with urethritis, 392 (43.3%) were infected with N. gonorrhoeae in the 2-year period 2013–2014. The mean age was 38.1 years (range, 18 to 71 years); 98.9% (388/392) were ethnic Han, and 69.6% (273/392) were married but indicated that they had had extramarital sex (an enrollment requirement in a separate study to examine the transmission of gonorrhea from men to women). All male subjects reported that they were heterosexual. Thirty-nine percent (153/392) reported that they had taken antibiotics in the 30 days prior to their clinic visit.

### Antimicrobial susceptibility testing.

**FIG 1:**
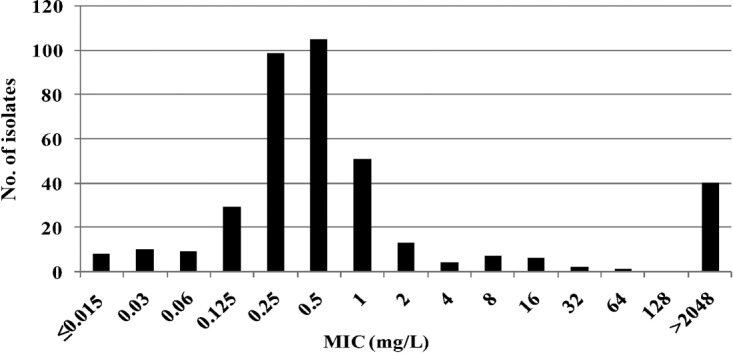
Distributions of MICs for AZM-R N. gonorrhoeae isolates in Nanjing from 2013 to 2014 (*n* = 384).

Of 392 isolates, 384 (98%) were recovered from storage. The MIC distribution of azithromycin for all isolates is shown in Fig. 1. The MICs of azithromycin ranged from ≤0.015 to >2,048 mg/liter, with a MIC_50_ and MIC_90_ of 0.5 and >2,048 mg/liter, respectively (Fig. 1). Of the 384 isolates, 124 (32.3%) displayed AZM-R, including 21.9% (84/384) with low- to mid-level resistance (MIC, 1 to 64 mg/liter) and 10.4% (40/384) that were highly resistant (defined as a MIC of ≥256 mg/liter but actually >2,048 mg/liter). Compared with that in 2008–2009, the prevalence of AZM-R isolates (6.8% [16/236]) (33) had increased significantly (*P* < 0.001) in the 5 intervening years. All AZM-R isolates were susceptible to spectinomycin and cefixime; however, coresistance to ciprofloxacin, tetracycline, and penicillin was also observed (100%, 91.1%, and 83.9%, respectively) (Table 1). In addition, 20 low- to mid-level AZM-R (MIC, 1 to 64 mg/liter) isolates also manifested decreased susceptibility to ceftriaxone (CRO-DS), similar to that reported for an isolate from a patient with a pharyngeal infection, who had become infected in Japan and failed treatment with ceftriaxone and azithromycin (37).

**TABLE 1:** Coresistance to ciprofloxacin, tetracycline, penicillin, spectinomycin, ceftriaxone, and cefixime among AZM-R N. gonorrhoeae isolates

Antimicrobial resistance^*a*^	No. (%) of AZM-R N. gonorrhoeae isolates (*n* = 124)
CIP-R	124 (100)
TET-R	113 (91.1)
PEN-R	104 (83.9)
SPT-R	0
CRO-DS	20 (16.1)^*b*^
CFM-DS	0

a CIP-R, TET-R, PEN-R, and SPT-R, resistance to ciprofloxacin, tetracycline, penicillin, and spectinomycin, respectively (MICs, ≥1, ≥2, ≥2, and ≥128 mg/liter, respectively). CRO-DS, and CFM-DS, decreased susceptibility to ceftriaxone and cefixime, respectively (MICs, ≥0.125 and ≥0.25 mg/liter, respectively).

b All 20 isolates exhibited low- to mid-level AZM-R.

### Determinants of resistance to azithromycin.

The AMZ-R mutation A2143G was found in the four alleles of the 23S rRNA gene in all HL-AZM-R isolates (*n* = 40) but was absent in all low- to mid-level AZM-R isolates (*n* = 84) (Tables 2 and 3). The C2599T mutation was present in the four 23S rRNA gene alleles in 59.5% (50/84) of isolates with low- to mid-level AZM-R but was absent from the 40 HL-AZM-R isolates (Tables 2 and 3).

**TABLE 2:** MICs, molecular resistance determinants, and NG-MAST types in 124 AZM-R N. gonorrhoeae isolates, Nanjing, 2013 to 2014

Azithromycin MIC (mg/liter) (no. of isolates)	23S rRNA mutation (no. of mutated alleles; no. of isolates)	*mtrR* mutation (no. of isolates)	NG-MAST ST (no. of isolates)
1–64 (84)	2599T (4; 50)	A-deletion in the *mtrR* promoter^*a*^ (70), *mtrR* A39T (4), *mtrR* G45D (11), *mtrR* H105Y (60)	3356 (7), 270 (5), 436 (5), 11931^*b*^ (4), 4007 (3), 568 (2), 1055 (2), 2318 (2), 11133 (2), 11890 (2), 11921^*b*^ (2), 11930^*b*^ (2), 11932^*b*^ (2), 11955^*b*^ (2), single STs (42)
≥256 (40)	A2143G (4; 40)	A-deletion in the *mtrR* promoter (36), *mtrR* A39T (1), *mtrR* G45D (28), *mtrR* H105Y (9)	1866 (18), 5309 (5), 5061 (4), 11929^*b*^ (2), single STs (11)

a A single nucleotide deletion (A) in the 13-bp inverted-repeat sequence of the *mtrR* promoter.

b New NG-MAST ST identified in this study.

**TABLE 3:** Comparison of gene mutations in low- to mid-level AZM-R versus HL-AZM-R N. gonorrhoeae isolates (*n* = 124)

Gene and presence or absence of resistance determinant(s)	No. (%) of N. gonorrhoeae isolates		*P* value^*a*^
	Low- to mid-level AZM-R (*n* = 84)	HL-AZM-R (*n* = 40)	
23S rRNA			
A2143G (all 4 alleles)	0	40 (100)	<0.001
No A2143G	84 (100)	0	
C2599T (all 4 alleles)	50 (59.5)	0	<0.001
No C2599T	34 (40.5)	40	
*mtrR*			
A-deletion in the promoter^*b*^			
A-deletion	70 (83.3)	36 (90.0)	0.325
No A-deletion	14 (16.7)	4 (10.0)	
Mutations in the coding region			
A39T	4 (4.7)	1 (2.5)	0.404
No A39T	80 (95.3)	39 (97.5)	
G45D	11 (13.1)	28 (70.0)	<0.001
No G45D	73 (86.9)	12 (30.0)	
H105Y	60 (71.4)	9 (22.5)	<0.001
No H105Y	24 (28.6)	31 (77.5)	

a Determined by the χ^2^ or Fisher exact test.

b A single nucleotide deletion (A) in the 13-bp inverted-repeat sequence of the *mtrR* promoter.

Examination of the *mtrR* promoter region showed that the prevalence of a single nucleotide deletion (A) in the 13-bp inverted-repeat sequence was no different in HL-AZM-R isolates (90% [36/40]) from that in low- to mid-level AZM-R isolates (83.3% [70/84]) (*P* > 0.05) (Tables 2 and 3). In contrast, the percentage (and number) of isolates with the G45D mutation in the *mtrR*-coding region was significantly higher among HL-AZM-R isolates (70% [28/40]) than among low- to mid-level AZM-R isolates (13.1% [11/84]) (*P* < 0.001). Significantly more low- to mid-level AZM-R isolates (71.4% [60/84]) than HL-AZM-R isolates (22.5% [9/40]) exhibited the H105Y mutation in *mtrR*-coding regions (*P* < 0.001). The prevalence of the A39T mutation did not differ between HL-AZM-R (2.5% [1/40]) and low- to mid-level AZM-R (4.7% [4/84]) isolates (Tables 2 and 3). Eighteen of 40 HL-AZM-R isolates were represented by a single N. gonorrhoeae multiantigen sequence typing (NG-MAST) type, ST1866 (see below). The prevalence of the G45D mutation was higher in the 18 ST1866 isolates (94.4% [17/18]) than in the remaining 22 HL-AZM-R isolates (50% [11/22]) (*P* < 0.05); the H105Y mutation was present in 41% (9/22) of the remaining 22 HL-AZM-R isolates but was absent from the 18 ST1866 isolates (*P* < 0.05). No isolates contained double mutations: A39T H105Y or G45D H105Y in the *mtrR* coding region. Point mutations in the *rplD* or *rplV* genes were not detected in any of the isolates tested.

### Molecular epidemiologic typing.

As determined by NG-MAST analysis, 71 different sequence types (STs) were represented in the 124 AZM-R isolates; 53/71 STs (74.6%) were represented once in single isolates (Table 2). Twenty-eight of the 71 STs represented (39.4%) have not been reported previously in the NG-MAST database. ST1866 predominated among the HL-AZM-R isolates (18/40 [45.0%]), followed by ST5309 (5 isolates), ST5061 (4 isolates), and ST11929 (2 isolates); 11 HL-AZM-R isolates each possessed a different ST. Among low- to mid-level AZM-R isolates, ST3356 (7 isolates) was the most prevalent ST, followed by ST270 (5 isolates), ST436 (5 isolates), ST11931 (4 isolates), and ST4007 (3 isolates); 9 different STs were represented in 9 pairs of low- to mid-level AZM-R isolates (9 groups of 2); 42 isolates each possessed a different ST.

**FIG 2:**
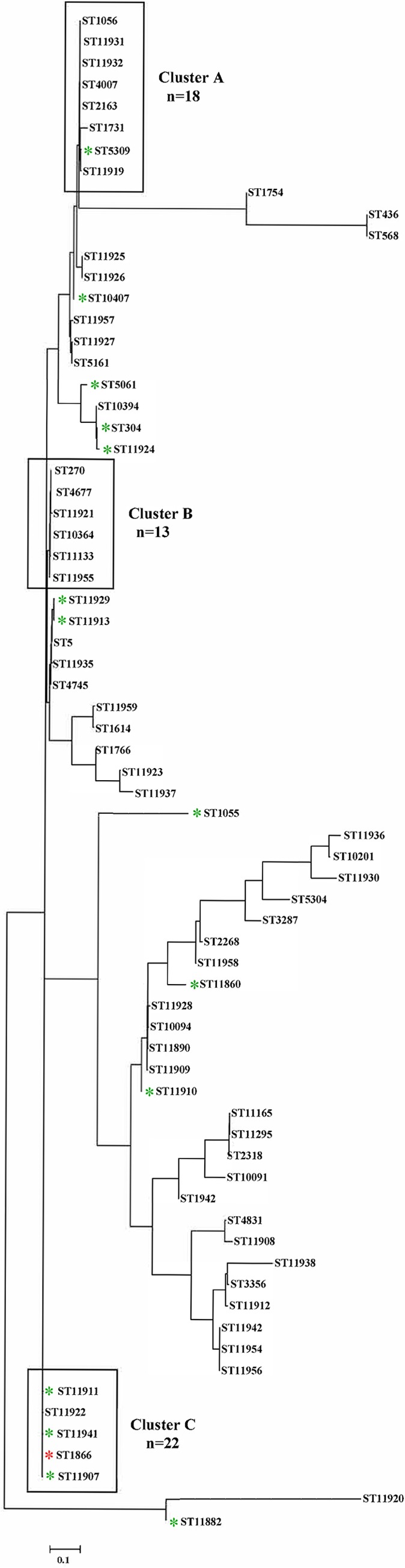
Phylogenetic tree constructed using MEGA7 with the concatenated sequences of *porB* and *tbpB* alleles for NG-MAST STs of 124 AZM-R N. gonorrhoeae isolates from Nanjing, China, 2013 to 2014. ST1866 is indicated by a red asterisk; non-ST1866, HL-AZM-R STs are indicated by green asterisks.

Three large clusters (>10 isolates) (boxed in Fig. 2) were identified based on phylogenetic analysis. Cluster A isolates were represented by 8 different STs with the same *tbpB* 186, except for ST5309 (*tbpB* 479); *tbpB* 186 and 479 differ by a single base pair only. Cluster B isolates were represented by 6 different STs; all were low- to mid-level AZM-R isolates and possessed the C2599T mutation in all four 23S rRNA gene alleles. Cluster C isolates were represented by 5 different STs. Notably, 4/5 STs (ST1866, ST11907, ST11911, and ST11941) in cluster C exhibited HL-AZM-R and possessed *tbpB* 33; each isolate contained the A2143G mutation in all four 23S rRNA gene alleles. The remaining ST in cluster C, ST11922, was associated with low- to mid-level AZM-R isolates possessing *tbpB* 215; *tbpB* 33 and 215 differ only by a single base pair. All 18 NG-MAST ST1866 isolates exhibited HL-AZM-R and were represented by two multilocus sequence types (MLSTs), MLST 10899 (*n* = 13) and MLST 12039 (*n* = 5). MLST 10899 and MLST 12039 share identical sequences in six of the seven housekeeping genes and contain only a single (different) SNP (single nucleotide polymorphism) in the *pdh* gene, which likely reflects ongoing evolution of the same strain. However, the remaining 22 HL-AZM-R isolates were widely distributed in the phylogenetic tree and did not belong to any single major cluster (Fig. 2).

### Prevalence of AZM-R N. gonorrhoeae in eastern Chinese cities.

**FIG 3:**
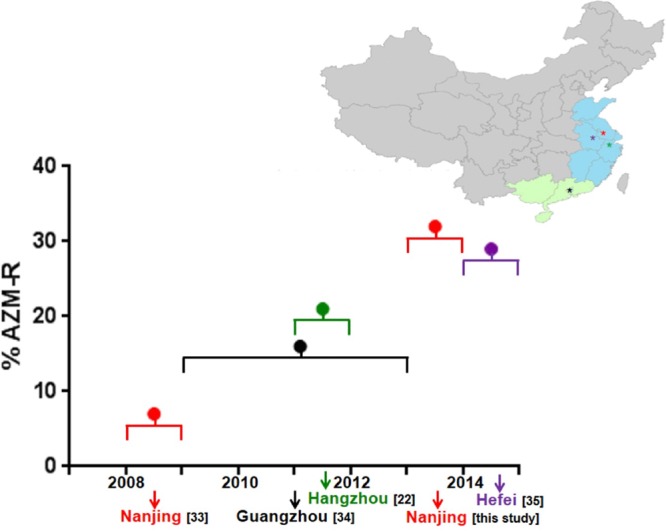
Geographic distribution and chronological emergence of reported AZM-R N. gonorrhoeae isolates in four Chinese cities. On the map, eastern China is shown in blue; southeastern China, in light green.

Four eastern/southeastern Chinese cities have reported AZM-R N. gonorrhoeae isolates between 2008 and 2015, and the prevalence of resistance has risen steadily (Fig. 3). In Nanjing (2008–2009), the prevalence of AZM-R N. gonorrhoeae was 6.8% (16/236) (33); in Hangzhou (2011–2012), resistance and high-level resistance to azithromycin were 21% (25/118) and 18% (21/118), respectively (22); in Guangzhou (2009–2013), resistance was 15.9% (77/485) (34); and in Hefei, resistance and high-level resistance to azithromycin were 28.6% (36/126) and 10% (13/126) in 2014 and 2015, respectively (35).

## DISCUSSION

AZM is widely used in China to treat C. trachomatis infection, but it is not recommended for use as monotherapy to treat gonorrhea. Instead, AZM is used in combination with primary antigonococcal therapy to treat coinfection with Chlamydia (11). Our results indicate that the prevalence of AZM-R N. gonorrhoeae in Nanjing has risen >4-fold, from 6.8% to 32.3%, in the 7-year period between 2008 and 2009 (33) to 2013–2014, exceeding the national average by ∼1 3/4-fold in the latter period (36). During an earlier and overlapping period between 2009 and 2015, AZM-R almost doubled in three other eastern (and southeastern) Chinese cities, progressing from 16% in Guangzhou (2009–2013) (34) to 21% in Hangzhou (2011–2012) (22) to 29% in Hefei (2014–2015) (35) (Fig. 3). However, contemporary reports indicate that AZM-R gonococcal infection outside China has remained low, e.g., 7.9% of isolates (163/2,069) in Europe in 2014 (12), 2.5% (127/5,093) in the United States in 2014 (13), and 1.5% (26/1,781) in South America in 2010 to 2011 (14).

Importantly, 18% of N. gonorrhoeae isolates in Hangzhou (22), 10% of isolates in Hefei (35), and 10.4% of isolates in the present Nanjing study exhibited high-level azithromycin resistance (HL-AZM-R), in contrast to the situation in Guangzhou, where between 2009 and 2013, HL-AZM-R was not detected (34). HL-AZM-R isolates have been documented in several countries, but most cases have been sporadic (15–21). In addition, our results show high coresistance to ciprofloxacin, tetracycline, and penicillin, findings that coincide with other reports (22, 38). Although HL-AZM resistance was not associated with gonococcal isolates that exhibited CRO-DS, the occurrence of 20 low- to mid-level AZM-R isolates with CRO-DS may threaten the effective use of dual antimicrobial treatment of gonorrhea, as evidenced by the reported failure to clear a gonococcal infection caused by an isolate with CRO-DS and low- to mid-level azithromycin resistance (37).

There are few studies that correlate clinical treatment failures with MICs for organisms that have failed treatment when AZM has been used as single therapy for gonorrhea; such reports rely on observations from clinical trials and case reports of treatment failures (39). One study (26), suggested that the MIC breakpoint associated with clinical treatment failures “appeared” to be 1 mg/liter when a 2-g dose of azithromycin was used alone to treat gonococcal urethritis in men, as evidenced by microbiologic cure in patients whose infecting strain had an AZM MIC of ≤0.25 mg/liter and microbiologic failure in 5 of 12 patients whose strain had a MIC of 1 mg/liter. A MIC of 1 mg/liter coincides with the azithromycin breakpoint for N. gonorrhoeae established by the European Committee on Antimicrobial Susceptibility Testing (EUCAST) (40). In our study, 32.3% of isolates had an AZM MIC of ≥1 mg/liter. Azithromycin was introduced into clinical practice in China in 1995 and has been widely used for respiratory, urogenital, dermatologic, and other infections. In China, azithromycin resistance has also been recognized in Treponema pallidum (41), Mycoplasma pneumoniae (42), Streptococcus pyogenes (43), and Mycoplasma genitalium (unpublished data). Cefixime has not been used for the treatment of gonorrhea in China, which may explain why all azithromycin-resistant isolates in our study were fully susceptible to cefixime. All strains were susceptible to spectinomycin, which is used widely in China for the treatment of gonorrhea. Spectinomycin MIC_50_ (16 mg/liter) and MIC_90_ (32 mg/liter) have remained unchanged for 10 years at the Nanjing STD clinic, and together with AZM for the treatment of C. trachomatis, spectinomycin may represent a second option for the treatment of gonorrhea in China (44, 45).

AZM exerts its bacteriostatic effect by interacting directly with the central loop of domain V of the *rrl* gene encoding the 23S rRNA, which results in blockage of protein synthesis. Specific point mutations that occur in this region likely lead to resistance by reducing the affinity of azithromycin for its target (28, 29). HL-AZM-R isolates harbor mutation A2143G in at least three of the four alleles (15–22); in our study, the A2143G mutation was present in all four alleles of HL-AZM-R N. gonorrhoeae. However, the A2143G mutation was absent in all of the low- to mid-level AZM-R isolates. The C2599T mutation, when present in two or more alleles, has been associated with low- to mid-level AZM-R isolates (21, 31, 38); in our study, 59.5% (50/84) of low- to mid-level AZM-R isolates possessed the C2599T mutation in all four alleles. Taken together, current studies (including ours) suggest that A2143G or C2599T mutations in the 23S rRNA alleles play a significant role in the mechanism of AZM resistance in gonococci (15–22, 34, 38).

Mutations in the promoter or coding sequence of the *mtrR* gene that are present in macrolide-resistant gonococcal strains can result in decreased expression of the MtrR repressor and consequent upregulation of the MtrCDE efflux pump (30, 31). In gonococcal strains with the A-deletion in the 13-bp inverted-repeat sequence of the *mtrR* promoter (which overlaps the *mtrCDE* promoter at the −35 region), *mtrR* expression is abrogated while *mtrCDE* expression is elevated, most likely because of the greater binding affinity of RNA polymerase for *mtrCDE* (46). A report of 59 gonococcal isolates from Japan indicated that the A-deletion in the *mtrR* promoter was significantly associated with MICs of ≥0.5 mg/liter (47), a finding similar to that in our study, where >80% of both HL-AZM-R and low- to mid-level AZM-R isolates possessed this A-deletion. Missense mutations in the *mtrR* gene that result in, for example, a G45D mutation in the helix-turn-helix motif (located at amino acid [aa] 32 to aa 53) in the MtrR repressor can diminish the binding of the repressor to the *mtrCDE* promoter (48, 49). In our study, the G45D mutation was present more often in HL-AZM-R isolates than in low- to mid-level AZM-R isolates (*P* < 0.001), a finding that coincides with previous findings (15, 22). The H105Y mutation in the MtrR repressor may inhibit MtrR dimerization and reduce binding to DNA (50). We showed that the H105Y mutation was more often present in low- to mid-level AZM-R isolates (*P* < 0.001), indicating that H105Y mutation could be implicated in the mechanism of low- to mid-level AZM resistance in N. gonorrhoeae. No point mutations were seen in the *rplD* or *rplV* genes, in agreement with another report (38).

NG-MAST analysis can be used to facilitate the tracking of antimicrobial-resistant strains. Chinese studies continue to identify new and diverse NG-MAST types (22, 33–35). In our study, 28/124 (22.6%) types were newly recognized; overall, 53/124 (42.7%) NG-MAST types were represented by single isolates. Eighteen of 40 HL-AZM-R isolates were represented by ST1866; the presence of the G45D and the H105Y mutations each differed significantly between the 18 ST1866 isolates and the 22 remaining HL-AZM-R isolates. Furthermore, phylogenetic analysis showed that the remaining 22/40 HL-AZM-R isolates were widely divergent and did not belong to any one of the major clusters, indicating high genetic variability of AMZ-R N. gonorrhoeae in more than half of the Nanjing isolates. As in Guangzhou (34), this may be related to large numbers of temporary residents in Nanjing, which results in a greater opportunity to import foreign (or new) NG-MAST STs. Because of the widespread use of azithromycin in China to treat patients with gonorrhea who are coinfected with C. trachomatis (and other bacteria) (33), we cannot exclude the possibility that antibiotic pressure and selection contributed to the diversity of NG-MAST types. With the exception of ST5309, which has also been reported from Australia (20), other NG-MAST STs represented in HL-AZM-R isolates in our study have not been reported in HL-AZM-R isolates from countries such as Argentina, which has reported ST696 (15), Scotland, reporting ST470 and ST649 (16), England, Wales, and the United States, reporting ST649 (19, 21), Ireland, reporting ST649 and ST3311 (17), Sweden, reporting ST285, ST332, and ST8727 (18), and Australia, reporting ST649, ST8917, ST10133, and ST10572 (20), suggesting that domestic sexual networks in Nanjing are not strongly linked to sexual networks abroad. NG-MAST ST1866 was the predominant ST (18/40 [45.0%]) among Nanjing HL-AZM-R isolates and was previously found in two AZM-R (MIC, >64 mg/liter) isolates in Nanjing in 2008–2009 (33) and, more recently, in six HL-AZM-R isolates from Hangzhou and 5 AZM-R isolates from Hefei, both eastern Chinese cities (22, 35). The 18 NG-MAST ST1866 HL-AZM-R isolates in our study had almost the identical MLST type (MLST 10899 and MLST 12039 differ only by one SNP in the *pdh* gene); a single MLST 10899 isolate was identified among 5 HL-AZM-R isolates in a report from Canada (51). Our study suggests that NG-MAST ST1866 isolates showing high-level azithromycin resistance may have been spreading in eastern China for many years.

In conclusion, a high prevalence of azithromycin resistance has emerged among N. gonorrhoeae isolates in Nanjing and other cities in eastern China. The A2143G mutation present in all four alleles of the 23S rRNA was associated with high-level azithromycin resistance (MIC, >2,048 mg/liter) and was identified in 10.4% (40/384) of Nanjing isolates of N. gonorrhoeae. The C2599T mutation present in all four alleles of the 23S rRNA was associated with low- to mid-level azithromycin resistance (MIC, 1 to 64 mg/liter) and was identified in 21.9% (84/384) of Nanjing isolates. NG-MAST and analysis of a phylogenetic tree revealed that two types of genetic signatures represent AZM-R strains in Nanjing: one is highly conserved among HL-AZM-R isolates (NG-MAST ST1866, representing 45% of HL-AZM-R isolates), and the other is highly diverse. These genotypes differ from those representing AZM-R strains in other parts of the world. Because 16.1% (20/124) of AMZ-R Nanjing isolates also manifest CRO-DS, the use of these two agents together for the treatment of gonorrhea in China, and indeed in other parts of the world, may become problematic.

## MATERIALS AND METHODS

### Gonococcal isolates and susceptibility testing.

Clinical gonococcal isolates were collected consecutively, between January 2013 and December 2014, from male adults with symptomatic urethritis (urethral discharge and/or dysuria) attending the sexually transmitted disease (STD) clinic at the National Center for STD Control in Nanjing, China. Isolates were cultured, identified, and preserved as described previously (52). MICs (mg/liter) of seven antibiotics (azithromycin, penicillin, tetracycline, ciprofloxacin, spectinomycin, cefixime, and ceftriaxone) were determined by the agar dilution method, according to the Clinical and Laboratory Standards Institute (CLSI) guidelines (53). For quality control, N. gonorrhoeae ATCC 49226, WHO reference strains A, G, J, K, O, P, and F, and a ceftriaxone-resistant strain (54) were included each time susceptibility testing was performed. AZM resistance was defined as a MIC of ≥1 mg/liter according to the European Committee on Antimicrobial Susceptibility Testing (EUCAST) guidelines (www.eucast.org) (40); susceptibility to other antibiotics was determined according to CLSI standards (53). Decreased susceptibility to ceftriaxone (CRO-DS) (MIC, ≥0.125 mg/liter) and to cefixime (MIC, ≥0.25 mg/liter) was defined by the WHO in 2012 (55). AZM-R N. gonorrhoeae isolates (MIC, ≥1 mg/liter) was divided into low- to mid-level (MIC, 1 to 64 mg/liter) and high-level (MIC, ≥256 mg/liter) resistance in accordance with previous studies (22, 34).

### Genetic determinants associated with resistance to azithromycin.

Genomic DNA was extracted using the DNA QuickExtract kit (Epicentre, USA) and was stored at −20°C. Primers and conditions used for PCR have been published previously (56, 57). All four alleles in the peptidyltransferase loop of domain V of the *rrl* gene encoding 23S rRNA were amplified using a two-step PCR and were sequenced as described previously (56). The *mtrR* gene and its promoter were also amplified by PCR and sequenced (57). PCR products were sequenced twice in both directions using an Applied Biosystems 3730xl DNA automatic sequencer. The nucleotide and deduced amino acid sequences were analyzed using the EditSeq program (LaserGene software [version 7.1; DNAStar Corp.]) and were aligned against their respective prototypes using the Megalign program (LaserGene software). To identify mutations, DNA sequences in the *rrl*, *mtrR*, *rplV*, and *rplD* genes were compared with corresponding sequences in N. gonorrhoeae reference strain FA1090 (GenBank accession no. AE004969.1), which is an antimicrobial-susceptible strain (pansensitive).

### Molecular epidemiologic typing.

Molecular epidemiologic analysis was performed on all AZM-R N. gonorrhoeae isolates by N. gonorrhoeae multiantigen sequence typing (NG-MAST), which assigns sequence types (STs) based on a combination of two hypervariable *porB* and *tbpB* alleles (58). Allele numbers and STs were assigned using the NG-MAST database (http://www.ng-mast.net). A maximum-likelihood phylogenetic tree was created using MEGA7 (http://www.megasoftware.net/download-form) (59) using concatenated *porB* and *tbpB* alleles. Eighteen NG-MAST ST1866 isolates with high-level AZM resistance were further typed using multilocus sequence typing (MLST) (60), which analyzes seven housekeeping genes (*abcZ*, *adk*, *aroE*, *fumC*, *gdh*, *pdh*, and *pgm*) (http://pubmlst.org/neisseria/).

### Review of AZM-R N. gonorrhoeae in eastern Chinese cities.

A Medline search was conducted using PubMed under the major headings of “Neisseria gonorrhoeae,” “azithromycin,” “China,”' and “antimicrobial resistance.”

### Statistical analysis.

A total of 124 AZM-R isolates were included in the statistical analysis. Chi-square (χ^2^) testing or the Fisher exact test was used to compare the number of isolates with resistance mutations in low- to mid-level AZM-R isolates with the number of high-level AZM-R (HL-AZM-R) isolates with mutations. IBM SPSS Statistics, version 19.0, was used for statistical analysis; a *P* value of ≤0.05 was considered statistically significant.
